# Efficient whole-cell catalysis for 5-aminovalerate production from L-lysine by using engineered *Escherichia coli* with ethanol pretreatment

**DOI:** 10.1038/s41598-020-57752-x

**Published:** 2020-01-22

**Authors:** Jie Cheng, Qing Luo, Huaichuan Duan, Hao Peng, Yin Zhang, Jianping Hu, Yao Lu

**Affiliations:** 1 Key Laboratory of Coarse Cereal Processing of Ministry of Agriculture and Rural Affairs, College of Pharmacy and Biological Engineering, Chengdu University, Chengdu, 610106 P. R. China; 2 College of Chemistry and Chemical Engineering, Yangtze Normal University, Chongqing, 408100 P. R. China; 3 College of Biological and Chemical Engineering, Guangxi University of Science and Technology, Liuzhou, 545006 P. R. China

**Keywords:** Biocatalysis, Metabolic engineering

## Abstract

Microorganisms can utilize biomass to produce valuable chemicals, showing sustainable, renewable and economic advantages compared with traditional chemical synthesis. As a potential five-carbon platform polymer monomer, 5-aminovalerate has been widely used in industrial fields such as clothes and disposable goods. Here we establish an efficient whole-cell catalysis for 5-aminovalerate production with ethanol pretreatment. In this study, the metabolic pathway from L-lysine to 5-aminovalerate was constructed at the cellular level by introducing L-lysine α-oxidase. The newly produced H_2_O_2_ and added ethanol both are toxic to the cells, obviously inhibiting their growth. Here, a promising strategy of whole-cell catalysis with ethanol pretreatment is proposed, which greatly improves the yield of 5-aminovalerate. Subsequently, the effects of ethanol pretreatment, substrate concentration, reaction temperature, pH value, metal ion additions and hydrogen peroxide addition on the whole-cell biocatalytic efficiency were investigated. Using 100 g/L of L-lysine hydrochloride as raw material, 50.62 g/L of 5-aminovalerate could be excellently produced via fed-batch bioconversion with the yield of 0.84 mol/mol. The results show that a fast, environmentally friendly and efficient production of 5-aminovalerate was established after introducing the engineered whole-cell biocatalysts. This strategy, combined with ethanol pretreatment, can not only greatly enhance the yield of 5-aminovalerate but also be applied to the biosynthesis of other valuable chemicals.

## Introduction

With the increasingly severe global environmental pollution and petroleum shortages, energy security has been widely concerned, and great efforts have been made to develop bio-based chemicals with renewable feedstock^1^. 5-aminovalerate (5AVA) is a non-proteinogenic amino acid that could be used as a potential, promising and valuable precursor for producing δ-valerolactam^2^, glutarate^3^ and nylon 5 fibers and resins^4^. It also could be used for synthesis of 5-hydroxyvalerate^5^, 1,5-pentanediol and other valuable chemicals. 5AVA could be manufactured from L-lysine (L-lys), which could be produced 2.2 million tons per year^3,6^. In view of the important applications of 5AVA in the field of synthesis, it is necessary to put forward relatively higher requirement for the development of the biotechnological 5AVA production.

In fact, the 5AVA production is closely related to the biological metabolism of L-lysine^7^. 5AVA is mainly synthetized through the coupled system of L-lys 2-monooxygenase (DavB) and 5-aminovaleramide amidohydrolase (DavA)^8^. Jorge *et al*. found that L-lys could be decarboxylated to form cadaverine by L-lys decarboxylase (CadA), then to 5AVA by consecutive transamination and oxidation^9^. Recently, Cheng *et al*. reported that L-lys hydrochloride (L-lys HCl) could be oxidative decarboxylated by L-lys α-oxidase (RaiP) from *Scomber japonicas* to produce 5AVA in *Escherichia coli* (*E. coli*)^10^. Based on these three basic synthetic routes converting L-lys to 5AVA, the three related bioproduction processes (i.e., microbial fermentation, enzymatic catalysis, and whole-cell methods) have been widely explored. For microbial fermentation, while DavA and DavB both were overexpressed in WL3110 and 3.6 g/L of 5AVA could be generated^11^. The engineered *Corynebacterium glutamicum* (*C. glutamicum*) strain under a synthetic H_36_ promoter could produce 33.1 g/L of 5AVA^12^. Putrescine transaminase (PatA), CadA and γ-aminobutyraldehyde dehydrogenase (PatD) were overexpressed in *C. glutamicum*, and 5.1 g/L of 5AVA was gained from glucose and alternative carbon sources^9^, as well as 28 g/L of 5AVA from a *de novo* bioproduction process with *gabT* gene deletion^13^. In Cheng’s research, the addition of 4% (v/v) ethanol effectively enhanced the level of RaiP protein expression, which could increase the titer of 5AVA to 29.12 g/L^10^. For the enzymatic catalysis of 5AVA, Liu *et al*. found that 20.8 g/L of 5AVA could be produced using the coupled system of purified DavB and DavA^5^. Under the catalysis of RaiP immobilized on a solid support, 13.4 g/L of 5AVA could be produced after 5 days^14^. In addition, an artificial iterative carbon-chain-extension cycle was constructed to generate 5AVA, a non-natural straight-chain amino acid^15^. Objectively, microbial fermentation has the disadvantages of long cycle, impure metabolites and complex process. The use of purified enzymes often leads to an increased production costs^16^.

Whole-cell biocatalysis has unique advantages, such as simple component of the reaction mixture, higher selectivity, highly catalytic activity and cost-effectiveness, etc. It has been widely used in the biosynthesis of 5AVA and other value-added chemicals^17^. In fact, whole-cell biocatalysis was considered as a competitive industrial process^18^, based on which Li *et al*. successfully obtained 24.8 g/L of β-alanine^19^. Cui *et al*. evaluated the effects of gene knockout on the production of glutathione by whole-cell catalysis^20^, and successfully prepared 103.1 g/L of 5AVA in the W3110/DavA-DavB^21^. Park *et al*. developed a novel process producing 90.59 g/L of 5AVA^22^. Li’s work showed that the overexpression of L-lys specific permease (LysP) enhanced 5AVA production, and 63.2 g/L of such compound could be successfully obtained using the whole-cell catalysis strategy^23^.

Bacterial strains often be engineered to express recombinant proteins. The expression of proteins is often induced by isopropyl β-D-thiogalactopyranoside (IPTG) in strain expression system. However, the expression level of proteins is still relatively low^24^. Effective strategies for improving expression level of proteins were developed, include the optimization of the host strains, vector, culture medium and gene sequences, as well as the addition of ethanol^25^. Ethanol has been demonstrated to works with T7 or T5 promoters, and a relatively higher level of protein expression was observed with the addition of 2%-4% ethanol^24^. In Chhetri’ research, the addition of 3% ethanol could not only improve the expression level of the heterogeneous amyloid-beta peptide^25^, but also increase the enzymatic activity by 5-fold^26^. Cheng *et al*. also found that the addition of 4% ethanol has a 3-fold increase in production of 5AVA^10^. Importantly, the maltose ABC transporter was revealed to be the main regulatory route activated to enhance the expression of heterologous proteins by ethanol^27^. Therefore, the ethanol pretreatment has proved to be a potential, environmentally friendly and efficient method to enhance the level of enzyme expression, and further to improve the yield of related reaction products^28^.

**Figure 1:**
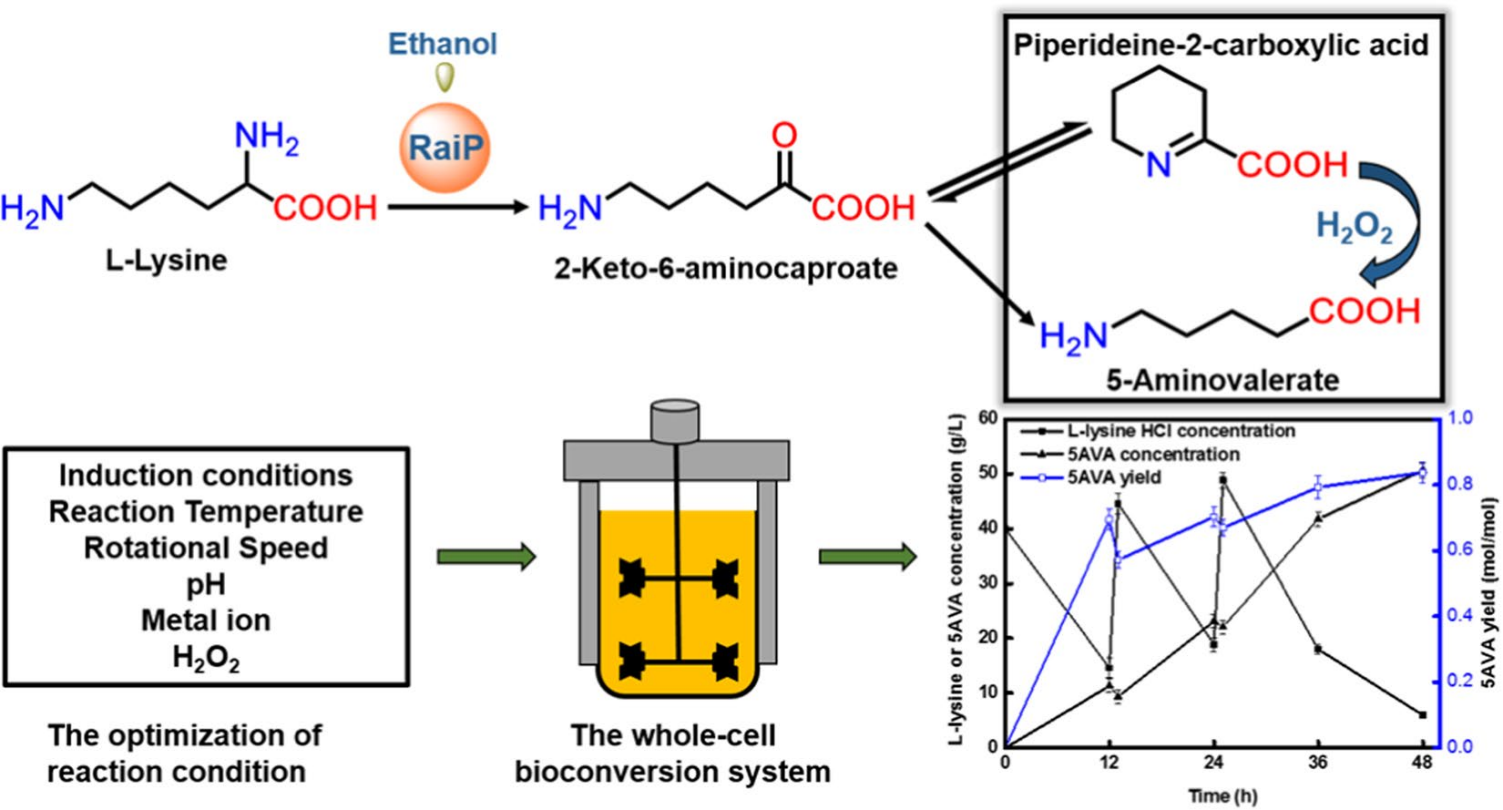
*E. coli* whole-cell overexpressing RaiP system with ethanol pretreatment converting L-lysine to 5-aminovalerate.

In this work, *E. coli* CJ02 overexpressed RaiP from *Scomber japonicas*used (CJ02RaiP) as whole-cell biocatalysts was constructed, sequentially followed by optimizing the concentration of ethanol pretreatment, reaction temperature, pH value, the additions of metal ion and hydrogen peroxide (see Fig. 1). Under optimal conditions, whole-cell biocatalysts CJ02RaiP exhibiting a better catalytic activity was finally achieved.

## Results and Discussion

### The effect of whole-cell catalyst with ethanol pretreatment on 5AVA production

In this work, two strategies—substrate L-lys HCl addition and *cadA* knockout—were conducted to increase the utilization of L-lys. Our previous studies have demonstrated that L-lys HCl is a better substrate to produce 5AVA^10^ and L-pipecolic acid (L-PA) than L-lys^29^, respectively increasing the titer of 5AVA^10^ by 24% and L-PA^29^ by 21%. Lys could be degraded to cadaverine by CadA^30^, which was knocked out to increase the flus to 5AVA. If not specified, L-lys was replaced by L-lys HCl in this work.

**Figure 2:**
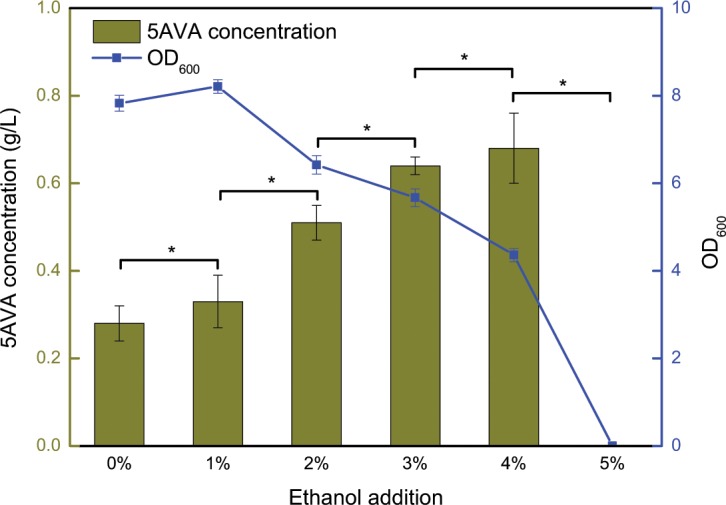
5AVA titer by whole-cell biocatalysts CJ02RaiP with ethanol pretreatment of different concentration at 37 °C, 250 rpm and pH 8.0 after 12 h. 5 g/L of L-lysine HCl was as substrate and the OD_600_ was 40. Various ethanol concentration of 0%, 1%, 2%, 3%, 4% and 5% were investigated. Data are means ± SD (n = 3). Statistics were performed by the two-tailed Student’s *t*-test. *p < 0.05; ns, not significant.

**Figure 3:**
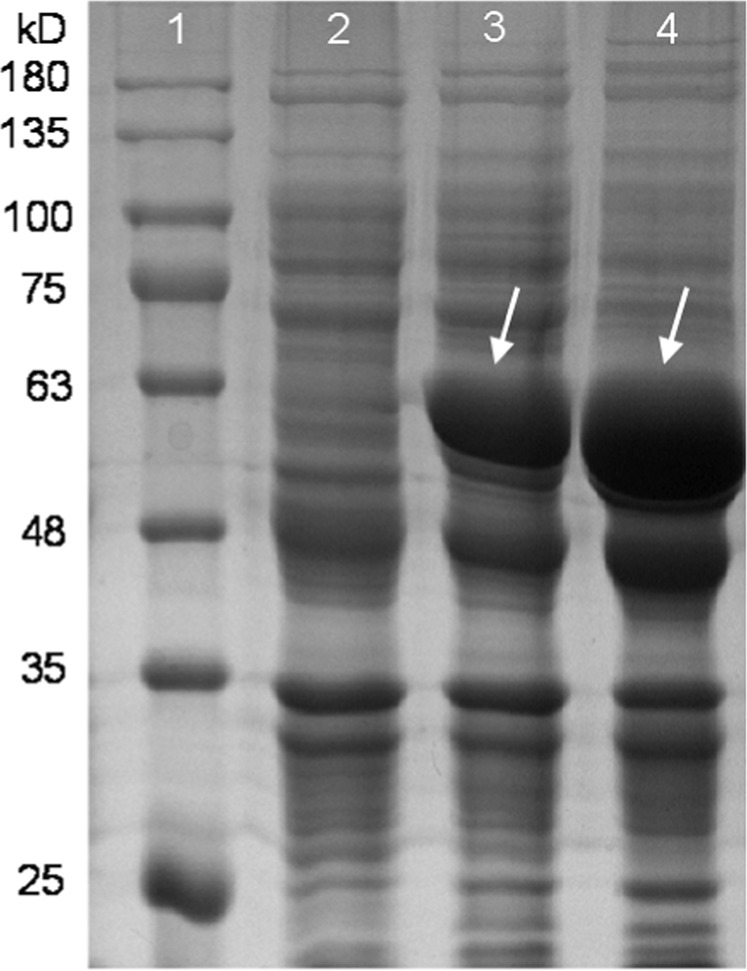
Expression analysis of RaiP. Protein samples were separated by 12% SDS-PAGE and stained with coomassie brilliant blue. Lane 1, molecular weight markers (kDa); Lane 2, RaiP noninduced control; Lane 3, RaiP induced in the absence of ethanol; Lane 4, RaiP induced in the presence of 4% ethanol. The expression was induced with 0.5 mM IPTG.

Figure 2 shows the effects of whole-cell biocatalysts CJ02RaiP on the production of 5AVA at different ethanol concentration of pretreatment, and other reaction conditions are 5 g/L of L-lys HCl, 37 °C and 250 rpm for 12 h (see Fig. 2). When the concentration of ethanol pretreatment was 1% (v/v), 0.33 g/L of 5AVA was produced after 12 h, which was 17.86% higher than that without ethanol pretreatment as control. When the concentration of ethanol pretreatment was increased to 2%, 3% and 4%, the titer of 5AVA reached 0.51 g/L, 0.62 g/L and 0.68 g/L, with the increase rates of 82.14%, 121.43% and 142.86%, respectively. However, when continuing to augment the concentration of ethanol pretreatment to 5%, the cell growth was unexpectedly significantly inhibited. Hence, the optimal concentration of ethanol pretreatment was set as 4%. It has been proved enhancing protein expression contributes to improving the titer of production^26,31^. Ethanol could also exhibit divergent effect on inclusion body formation^32^. As shown in Fig. 3, the addition of 4% ethanol increased the expression of RaiP protein by about 30% compared with the absence of ethanol. In this work, whole-cell catalysts with ethanol pretreatment did improve the production of 5AVA significantly.

### Optimization of whole-cell transformation conditions

#### Reaction temperature

**Figure 4:**
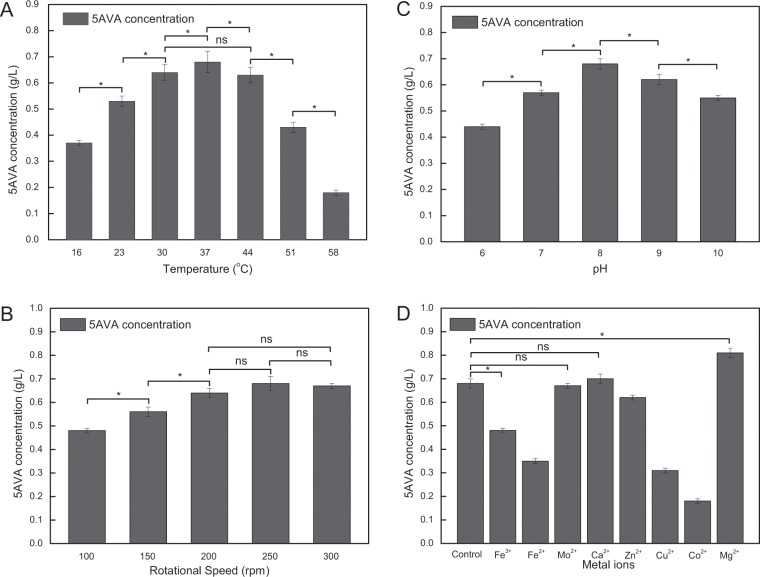
(**A**) Biosynthesis of 5AVA achieved via whole-cell biocatalysts CJ02RaiP with 4% ethanol pretreatment at different temperature, 5 g/L L-lysine HCl, 250 rpm and pH 8.0 for 12 h. Various temperature of 16 °C, 23 °C, 30 °C, 37 °C, 44 °C, 51 °C and 58 °C were investigated. (**B**) Biosynthesis of 5AVA achieved via whole-cell biocatalysts CJ02RaiP with 4% ethanol pretreatment at different rotational speed, 5 g/L L-lysine HCl, 37 °C and pH 8.0 for 12 h. Various rotational speed of 100 rpm, 150 rpm, 200 rpm, 250 rpm and 300 rpm were investigated. Data are means ± SD (n = 3). Statistics were performed by the two-tailed student t-test. *p < 0.05; ns, not significant. C: Biosynthesis of 5AVA achieved via whole-cell biocatalysts CJ02RaiP with 4% ethanol pretreatment at different pH, 5 g/L L-lysine HCl, 37 °C and 200 rpm for 12 h. Various pH of 6, 7, 8, 9 and 10 were investigated. D: The effect of metal ions on 5AVA production were investigated with 4% ethanol pretreatment at 5 g/L L-lysine HCl, 37 °C, pH 8.0 and 200 rpm for 12 h. Various metal ions of Mg^2+^, Mo^2+^, Ca^2+^, Zn^2+^, Fe^2+^, Fe^3+^, Cu^2+^ and Co^2+^ (3 mM) were investigated. Data are means ± SD (n = 3). Statistics were performed by the two-tailed Student’s *t*-test. *p < 0.05; ns, not significant.

To enhance the biocatalytic efficiency, the optimal reaction temperature was discussed here. Figure 4A shows the 5AVA production catalyzed by whole-cell biocatalysts CJ02RaiP with 4% ethanol pretreatment at different temperature, 5 g/L of L-lys HCl, pH 8.0 and 250 rpm for 12 h. It was shown that the reaction temperature ranging from 16 to 58 °C had obviously influence on 5AVA production. The whole-cell biocatalysts CJ02RaiP could only generate 0.37 g/L of 5AVA at 16 °C. As the reaction temperature increased, the titer of 5AVA increased gradually. When the reaction temperature was 37 °C, the titer of 5AVA was 0.68 g/L. Notably, only 0.18 g/L of 5AVA was obtained at 58 °C, implying that the whole-cell catalytic activity decreased to very low levels at high temperature. The results were consistent with the character of most enzymes, that is, the function curve of enzyme activity to temperature is an inverted bell shaped. Hence, 37 °C was set as the optimal reaction temperature.

#### Rotational speed

The whole-cell catalysis was executed in the flasks with rotation, allowing the permeation of the oxygen in the air. Here, O_2_ is the electron acceptor of the oxidative, and NH_3_ is produced as a byproduct for enzyme RaiP to transform L-lys HCl to 5AVA. Obviously, rotational speed could partly determine the production of 5AVA by affecting the dissolved oxygen. Figure 4B shows the 5AVA production performed at different rotational speed, 5 g/L of L-lys HCl, pH 8.0 and 37 °C for 12 h. With the increase of rotational speed, the titer of 5AVA is slightly increased. When the stirring rate was 100 rpm, the titer of 5AVA reached 0.48 g/L; when the rate become 200 rpm, the corresponding titer increased to 0.64 g/L; as the rotation speed continued to increase, there was no significant difference in the titer of 5AVA. Thus, 200 rpm was adopted in the subsequent experiments.

#### pH value

To achieve a higher titer of product, the pH values during the 5AVA production were also discussed. The relevant reaction conditions were the 50 mM PKB buffer containing 5 g/L of L-lys HCl, the pH values ranged from 6.0 to 10.0, reaction temperature of 37 °C, culture time of 12 h at 200 rpm shaking. As shown in Fig. 4C, the optimal pH value for production of 5AVA was 8.0. It can be seen that the conditions of excessive acid and alkali are not conductive to the bioconversion from L-lys HCl to 5AVA, thus the subsequent experiments were executed at pH value of 8.0.

#### Metal ions

Figure 4D shows the influence of metal ions on the 5AVA production in whole-cell biocatalysts CJ02RaiP with 4% ethanol pretreatment. As shown in Fig. 4D, the addition of 3 mM Mg^2+^ exhibited a positive effect on the 5AVA production with 0.19-fold higher, and the titer of 5AVA increased from 0.68 to 0.81 g/L. There is no significant variation in the catalytic activity by adding Mo^2+^, Zn^2+^ or Ca^2+^, while the addition of Fe^3+^, Fe^2+^, Co^2+^ or Cu^2+^ exhibited negative influence. Specially, the Cu^2+^ and Co^2+^ both significantly decreased the catalytic activity of whole-cell biocatalysts CJ02RaiP (see Fig. 4D). Nevertheless, the addition of Mg^2+^ could significantly improve the activity of whole-cell catalysts CJ02RaiP. Positive effect of Mg^2+^ was reported in phosphoenolpyruvate carboxylase^33^, malate dehydrogenase^34^ or pyruvate carboxylase^35^, where exogenous Mg^2+^ ion addition enhanced the enzyme activity to produce succinate. As a substrate activator^36^, Mg^2+^ might be associated with organic cofactors to form stable units like our study. However, in a previous study, the addition of Zn^2+^ could also improve the enzyme activity about 1.1-fold at the concentration of 1 mM^37^. It is speculated that low concentration (1 mM) of Zn^2+^ could improve the activity of RaiP, whereas high concentration (3 mM) inhibited its enzyme activity.

### The effect of H_2_O_2_ as oxidant on the 5AVA production

**Figure 5:**
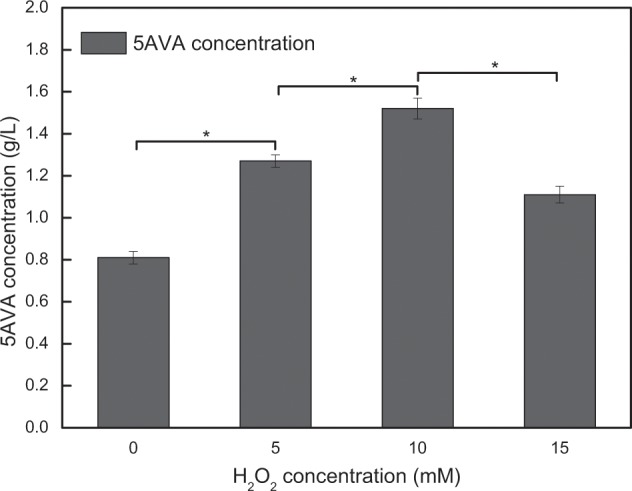
5AVA production achieved by engineered whole-cell biocatalysts CJ02RaiP in the presence of different H_2_O_2_ concentrations. The experiments were conducted at 5 g/L L-lysine HCl, 4% ethanol pretreatment, 37 °C, 3 mM Mg^2+^, pH 8.0 and 200 rpm for 12 h. Various H_2_O_2_ concentrations (0, 5, 10 and 15 mM) were added after reaction 8 h. Values and error bars represent the mean and the standard deviation of triplicate cultivations. Statistics were performed by the two-tailed Student’s *t*-test. *p < 0.05; ns, not significant.

The addition time of H_2_O_2_ was also optimized in this study. 1.27 g/L of 5AVA could be achieved by adding 5 mM H_2_O_2_ after 8 h reaction, increasing by 19.81% compared with 0 h. However, the titer of 5AVA was decreased if H_2_O_2_ continued to be added later. Figure 5 shows the effects of H_2_O_2_ on the 5AVA production by the whole-cell biocatalysts CJ02RaiP with 4% ethanol pretreatment. After the addition of 5 mM H_2_O_2_, whole-cell catalysts CJ02RaiP could produce 1.27 g/L of 5AVA after 12 h, which increased by about 66.67% compared with the absent of H_2_O_2_ addition. When 10 mM H_2_O_2_ was added, the titer of 5AVA significantly increased to 1.52 g/L, with a 5AVA titer increase of 87.65%. The results indicated that the addition of H_2_O_2_ could favor the reaction to 5AVA and increase its metabolic flux. When continuing to increase the concentration of H_2_O_2_ addition, the titer of 5AVA was decreased. High concentration of H_2_O_2_ might reduce the enzyme activity of RaiP. It is in accordance with the previous reported findings of Cheng *et al*., where a higher concentration of 5AVA could be obtained by adding H_2_O_2_ after fermentation for 8 h^10^. High concentration of H_2_O_2_ could significantly inhibit enzyme activity of RaiP in this work. Hence, we proposed a strategy that high concentration of H_2_O_2_ was added after 8 h reaction of CJ02RaiP helping to accumulate intermediate 2-keto-6-aminocaproate (2K6AC) in the biocatalytic process. In order to eliminate the negative effects in the growth process, adding H_2_O_2_ as oxidant at the later stage of growth could be an alternative method.

### Whole-cell catalysis and sale-up bioconversion of 5AVA production

**Figure 6:**
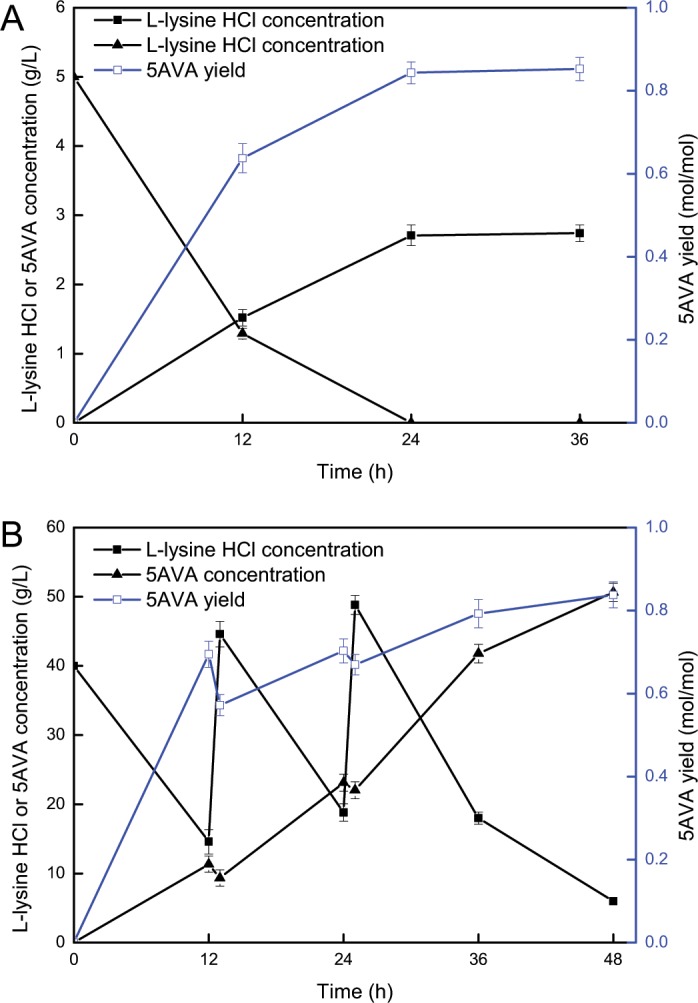
(**A**) Time profiles of 5AVA production were investigated by engineered whole-cell biocatalysts CJ02RaiP. The experiments were conducted at 5 g/L L-lysine HCl, 4% ethanol pretreatment, 37 °C, 3 mM Mg^2+^, pH 8.0 and 200 rpm. 10 mM H_2_O_2_ was added after reaction 8 h. Values and error bars represent the mean and the standard deviation of triplicate cultivations. (**B**) Fed-batch strategy for the production of 5AVA by engineered whole-cell biocatalysts CJ02RaiP in a 400 mL reactor. The experiments were conducted at 100 g/L L-lysine HCl, 4% ethanol pretreatment, 37 °C, 3 mM Mg^2+^, pH 8.0 and 200 rpm. 10 mM H_2_O_2_ was added after reaction 8 h. Data are means ± SD (n = 3).

The whole-cell biocatalysis from L-lys HCl to 5AVA was conducted under the optimal conditions to investigate the feasibility of CJ02RaiP as a 5AVA producer in a 2 mL reactor supplemented with 5 g/L of L-lys HCl. The whole-cell biocatalyst CJ02RaiP could consume more than 60% of the L-lys HCl after 8 h (see Fig. 6A). After 24 h, the reaction went to completion and 2.71 g/L of 5AVA was produced. The yield of 5AVA was greatly improved during the biocatalytic process (see Fig. 6A), due to that L-lys HCl was firstly converted into intermediate 2K6AC catalyzed by RaiP, and then oxidatively decarboxylated into 5AVA in the presence of the oxidant H_2_O_2_.

To further examine the industrial potential of CJ02RaiP, a fed-batch strategy was adopted in a 400 mL reactor to enhance the 5AVA production. 11.35 g/L of 5AVA was produced after 12 h (see Fig. 6B), where 30 g/L of L-lys HCl was added into the reactor to maintain high-efficiency bioconversion; 5AVA titer increased to 23.12 g/L at 24 h; 50.62 g/L of 5AVA with a yield of 0.84 mol/mol was obtained at 48 h by adding another 30 g/L of L-lys HCl. At this point, the production of 5AVA reached equilibrium, no obvious increases in 5AVA production were obtained after 48 h. At the end of the reaction, there were 6.05 g/L of Lys HCl and a little intermediate 2K6AC remained.

Whole cell catalysis could usually obtain a higher yield^17^. 63.2 g/L of 5AVA was produced by whole cell catalysis with a yield of 0.75 g/g^23^. Compared with microbial fermentation in Cheng’s study^10^, whole-cell catalysis could produce a higher titer from 29.12 to 50.62 g/L, and shorten the reaction time from72 to 48 h. In addition, the produced H_2_O_2_ and added ethanol during the fermentation process markedly inhibited the cell growth, resulting in the highest OD_600_ of only 41^10^. Satisfactorily, whole-cell catalysis is free of cell growth restrictions, and OD_600_ could reach up to 80, thus further improving 5AVA production. In fact, the high yield of 5AVA is related the two-step reaction mechanism: (1) the intermediate 2K6AC was heavily accumulated by RaiP; (2) hydrogen peroxide served as oxidant to form 5AVA through decarboxylation and oxidation.

## Conclusions

A green whole-cell biocatalytic production for the 5AVA was established in this work. CJ02RaiP, as an efficient whole-cell biocatalyst, could produce 0.81 g/L of 5AVA from 5 g/L of L-lys HCl with 4% ethanol pretreatment after reaction 12 h. The yield of 5AVA production with ethanol pretreatment was 189.29% higher than that without pretreatment. A titer of 1.52 g/L of 5AVA was obtained after reaction 12 h using whole-cell biocatalysts CJ02RaiP with 4% ethanol pretreatment and 10 mM H_2_O_2_, which increased 5AVA production by 87.65% than that in the absence of H_2_O_2_. After a series of process optimizations (i.e., pretreatment with 4% ethanol, and the addition of 10 mM H_2_O_2_ after reaction 48 h, etc.), 50.62 g/L of 5AVA can be obtained based on this engineered whole-cell biocatalytic system. The constructed whole-cell biocatalyst system with ethanol pretreatment shows the advantages of renewable substrate, simple component of the reaction mixture, higher titer and environmental friendliness. It has important application values for large-scale production of 5-aminovalerate and other valuable chemicals.

## Materials and Methods

### Microorganisms and culture conditions

*E. coli* DH5α was used for the propagation of plasmids, *E. coli* BL21 (DE3) and its derivative both used for 5AVA production, and vector pET21a for protein expression. The nucleotide sequence of the synthetic and optimized *raiP* gene is available in the GenBank database (Refseq accession No. MG423617)^29^. The *raiP* from *Scomber japonicas* with *Bam*HI and *Nde*I restriction sites was ligated into the vector pET21a to form pCJ01. The gene *cadA* was knocked out in *E. coli* BL21 (DE3) to obtained strain ML03 using the two steps of homologous^10^. The strain ML 03 harboring plasmid pCJ01 was named CJ02. All the resulting strains were cultured in Lysogeny broth (LB) medium supplemented with corresponding antibiotics.

### Preparation of the whole-cell biocatalysts with ethanol pretreatment

*E. coli* ML03 harboring plasmid pCJ02 was grown at 37 °C for 12 h with 250 rpm shaking in 2 L flask containing 240 mL LB with 100 μm/mL ampicillin. When the optical density (OD_600_) of 0.6 was reached, ethanol and 0.5 mM IPTG both were added, and the culture was continued at 20 °C for 16 h. In order to improve the biocatalytic efficiency, the concentrations of ethanol pretreatment were also studied. The influence of the different concentrations (i.e., 1%, 2%, 3%, 4% and 5%) of ethanol pretreatment on 5AVA production were discussed. Moreover, the effects of different types of metal additions (i.e., Fe^2+^, Mn^2+^, Mg^2+^, Mo^2+^, Co^2+^, Ca^2+^, Zn^2+^, Cu^2+^ and Fe^3+^ at constant concentrations of 3 mM) on yield were also investigated.

### Conditions and scale-up of bioconversions

For 5AVA bioconversion, the cells were firstly harvested by centrifugation at 10,000 rpm for 10 min after 16 h culture, then washed with potassium phosphate buffer (PKB, 50 mM, pH 8.0) twice, and then resuspended in 2 mL (OD_600_ = 40) reaction mixture containing 5 g/L of L-lys HCl. The bioconversion reactions were performed at 37 °C and pH 8.0, with 250 rpm shaking for 12 h. For the scale-up of bioconversion, the cell culture was concentrated, and suspended in 400 mL (OD_600_ = 80) PKB buffer with 40 g/L of L-lys HCl. Other reaction conditions include: 25% NH_4_OH was used to adjust the pH value to 8.0; aeration rate was set as 1 vvm; 30 g/L of L-lys HCl was added after 12 h bioconversion; additional L-lys HCl was added after 24 h bioconversion.

### Analytical procedures

**Figure 7:**
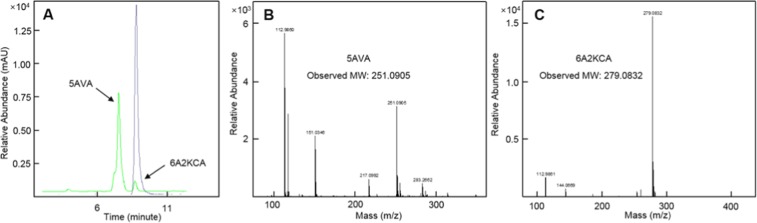
LC-MS confirmation of 5AVA and 2K6AC biosynthesis by whole-cell biocatalysts CJ02RaiP. (**A**) HPLC results of 5AVA and 2K6AC. (**B**) Mass spectrum of 5AVA. (**C**) Mass spectrum of 2K6AC. Samples were derived with phenyl isothiocyanate (PITC) for LC-MS analysis. 5AVA, 5-Aminovalerate. 2K6AC, 2-Keto-6-aminocaproate.

The optical density was determined by using an Ultrospec^TM^ 2100 pro UV/visible spectrophotometer (USA). Extracellular L-lys and 5AVA were quantified using a high performance liquid chromatography (HPLC) system (1260 series, Aglient Co., Ltd, CA, USA) equipped with a UV detector. To monitor the concentrations of 5AVA and L-lys, the method of phenyl isothiocyanate (PITC), described in our previous article, was adopted in this work^10^. Quantitative determination of 2K6AC was not performed due to the lack of its standard substrate. For liquid chromatography-mass spectrometry (LC-MS) identification of 5AVA and 6A2KCA, exact mass spectra was explored with a Bruker micrOTOF-Q II mass spectrometer, using the time of flight (TOF) technique as well as equipped with an ESI source operating in negative mode (Burker Co., Ltd, USA). The intermediate 2K6AC and 5AVA were qualitatively verified by LC-MS (see Fig. 7). As shown in Fig. 7A, the approximate retention time of 5AVA and 2K6AC was 7.2 and 8.9 min, respectively. According to Fig. 7B,C, the [M-H]^-^ of 5AVA and 2K6AC were 251.0905 and 279.0832, respectively.

## Data Availability

The datasets generated during the current study are available from the corresponding author on reasonable request.
